# National Medical Convention

**Published:** 1846-06-01

**Authors:** 


*National Medical Convention.*—The National Medical Convention, was held on the 5th ult., at the University Medical College, at New York, agreeably to appointment by the State Medical Society. About one hundred Delegates, representing sixteen States were present. After a session of two days, having appointed Committees to report on various subjects at another Convention, to be held in May next, at Philadelphia, the meeting was adjourned *sine die*. It was voted to publish a large edition of the transactions, and we have made arrangements to be supplied with a sufficient number of copies, to send one to each of our subscribers. If they arrive in season, they will accompany this number; if not, they will be sent as soon as they are received. This will spare us the necessity of devoting any of our space to a detailed account of the proceedings.

We defer comments on certain matters connected with the Convention, which afford occasion for remarks; and for the present confine ourselves to a general expression of satisfaction with the succcess of the undertaking, and the results attained. Although not so completely national as could have been wished, it was as much so as could reasonably have been expected, and in the amount of aggregated character and ability, was all that could be desired. We confess that we experienced emotions of pride and satisfaction, as we surveyed the countenances of our brethren, gathered from distant portions of our country, nor were these emotions diminished by their deliberations, which, as a whole, were characterized by dignity, harmony, and good feeling. They, however, who had not the opportunity to judge from personal observation of the character of the Convention, need not the testimony of those who were present, since a large proportion of its members are well know by merited professional distinction.

It may appear to some, that very little was accomplished. So it does not seem to us. It was not a little to assemble so large and respectable a delegation. It demonstrated the fact, which not a few had doubted, that the plan of national organization for the objects proposed, is practicable. Had it been generally believed throughout our country that the Convention would have been in numbers and character what it was, the representation would doubtless have been more complete. Uncertainty as to the result, we know, deterred many associations from sending delegates. This obstacle will not exist in the way of the next Convention, which we firmly believe will embrace a full representation from every state in the Union.

To consider this as but preliminary to another and more complete Convention, in our view, was not only judicious, but absolutely necessary for the successful accomplishment of the objects desired. In order for the action taken by a Medical Congress to succeed in harmonizing different opinions and interests, and be received with a binding force by the majority of the Profession, the subjects coming before it must be carefully investigated, all the facts bearing upon them collected, and the various considerations which are involved carefully weighed, previously to a final decision being called for. These conditions could not be complied with at a first, short session, even had the Convention been sufficiently complete. A year's delay, will afford time for reflection and discussion, and the formation of deliberate and settled opinions by the Profession at large : and in the mean time, it is to be expected that the several Committees appointed, will obtain all the data necessary for a full understanding of the different topics upon which it is their duty to report. The next Convention will therefore avoid the imputation of acting prematurely, and will be prepared to arrive at conclusions which will be far more likely to meet with general approbation and concurrence. And beside, the next Convention will be more truly and emphatically national in its composition, and its results will consequently have far greater influence and authority.

So far as we could judge, the course pursued by the Convention was almost unanimously acceptable to its members, and all seemed to separate satisfied with what had been accomplished, and with full confidence that the incipient steps had been taken in a movement which will be of immense importance to the character of the Medical Profession of our country.

We shall continue, as heretofore, to devote a share of our editorial and eclectic department, to matters relating to the topics which will come before the Convention of 1847 ; and we invite free discussion of these topics through the medium of our columns.

